# Aligning marine species range data to better serve science and conservation

**DOI:** 10.1371/journal.pone.0175739

**Published:** 2017-05-03

**Authors:** Casey C. O'Hara, Jamie C. Afflerbach, Courtney Scarborough, Kristin Kaschner, Benjamin S. Halpern

**Affiliations:** 1 National Center for Ecological Analysis and Synthesis, University of California, Santa Barbara, CA, United States of America; 2 Department of Biometry and Environmental Systems Analysis, Albert-Ludwigs University, Tennenbacher Straße 4, Freiburg i. Br., Germany; 3 Bren School of Environmental Science and Management, University of California, Santa Barbara, CA, United States of America; 4 Department of Life Sciences, Imperial College London, Silwood Park Campus, Ascot, United Kingdom; University of Colorado, UNITED STATES

## Abstract

Species distribution data provide the foundation for a wide range of ecological research studies and conservation management decisions. Two major efforts to provide marine species distributions at a global scale are the International Union for Conservation of Nature (IUCN), which provides expert-generated range maps that outline the complete extent of a species' distribution; and AquaMaps, which provides model-generated species distribution maps that predict areas occupied by the species. Together these databases represent 24,586 species (93.1% within AquaMaps, 16.4% within IUCN), with only 2,330 shared species. Differences in intent and methodology can result in very different predictions of species distributions, which bear important implications for scientists and decision makers who rely upon these datasets when conducting research or informing conservation policy and management actions. Comparing distributions for the small subset of species with maps in both datasets, we found that AquaMaps and IUCN range maps show strong agreement for many well-studied species, but our analysis highlights several key examples in which introduced errors drive differences in predicted species ranges. In particular, we find that IUCN maps greatly overpredict coral presence into unsuitably deep waters, and we show that some AquaMaps computer-generated default maps (only 5.7% of which have been reviewed by experts) can produce odd discontinuities at the extremes of a species’ predicted range. We illustrate the scientific and management implications of these tradeoffs by repeating a global analysis of gaps in coverage of marine protected areas, and find significantly different results depending on how the two datasets are used. By highlighting tradeoffs between the two datasets, we hope to encourage increased collaboration between taxa experts and large scale species distribution modeling efforts to further improve these foundational datasets, helping to better inform science and policy recommendations around understanding, managing, and protecting marine biodiversity.

## Introduction

Knowing where species exist and thrive is fundamental to the sciences of ecology, biogeography, and conservation. This body of science has resulted in various compiled databases of species distribution and range maps which provide foundational information for understanding species diversity and extinction risk, predicting species responses to human impacts and climate change, and managing and protecting species effectively. No species range dataset can claim to represent the "truth" of any species' spatial distribution, but rather each offers its own distinct understanding of that latent distribution. These varying predictions of species presence and absence, driven by intent and methodology, can result in very different predictions of species' ranges. However, conservation managers and policy makers must base their actions on conclusions drawn from these imperfect datasets. It is critical to understand the differences in spatial range datasets and the implications of these differences for conservation research and decision-making.

A broad literature exists on methods and results for predicting individual species distributions, but relatively few have been applied to broad suites of taxa globally, particularly in marine contexts. The two most comprehensive, widely-used global-scale repositories that predict marine species ranges throughout the world's oceans are AquaMaps, which rely primarily on model predictions to communicate the distribution of a species based on habitat suitability [1], and range data from the International Union for Conservation of Nature (IUCN), which rely primarily on expert opinion to communicate range size as a criterion for extinction risk assessments [2].

While the two datasets ostensibly describe the same information, i.e., where can a particular species be found, they communicate the information in very different ways. The IUCN range map for a given species describes the "extent of occurrence" for that species, an undifferentiated contiguous region that encloses all known occurrences and connecting regions, with the explicit caveat that this "does not mean that [the species] is distributed equally within that polygon or occurs everywhere within that polygon" [2]. The AquaMaps map for a species, on the other hand, provides a more nuanced understanding of species distribution by attempting to estimate a “relative probability of occurrence” of a species throughout its predicted range based on information about species-specific habitat usage [1]. By considering any non-zero probability of occurrence, we can effectively consider AquaMaps as an extent of occurrence, but one that is bounded by modeled environmental conditions instead of being delineated manually by experts.

The fundamental differences between the datasets suggest that the choice of one over the other (and for AquaMaps, the consideration of a presence threshold) should be carefully matched to the purpose for which it is to be used [3], and yet these datasets have been used in many studies and applications for a wide range of purposes, typically without directly addressing the limitations of the chosen dataset or comparing outcomes between the two. Example uses of the data include assessing marine species status [4–6], evaluating global biodiversity patterns [7–11], predicting species range shifts [12], and setting conservation priorities [13].

We recognize that each dataset provides distinct value for conservation research, and so here we focus not on how the two datasets should ideally be used, but instead, on hidden assumptions and sources of error within each dataset. Importantly, we test the implications of choosing one over the other, and use overlapping species mapped within both datasets to better understand the causes of differences in results from using one or the other. We also repeat a recent analysis of gaps in protection afforded by marine protected areas (MPAs) to examine the implications of these differences for global conservation and management priorities. Because these datasets are so widely used, it is crucial to understand and acknowledge, and where possible address, data limitations.

## Methods and analysis

### About the datasets

The IUCN has published species range maps developed by species experts for 4,027 unique marine species. Experts outline spatial boundaries that define the "limits of distribution" of a given species, based on observation records and informed by expert understanding of species' range and habitat preferences (S1A Fig). IUCN guidelines recommend that boundaries be drawn as a "minimum convex polygon", i.e., "the smallest polygon in which no internal angle exceeds 180 degrees and which contains all the sites of occurrence" [2].

In contrast, AquaMaps has modeled species distribution for 22,889 species based on species-specific envelopes of environmental preference, such as temperature, depth, and salinity. These preference envelopes are deduced from occurrence records and published species databases such as FishBase [14] and SeaLifeBase [15], and can be further revised based on expert knowledge about species-specific habitat usage. The AquaMaps model overlays these environmental preferences atop a map of environmental attributes on a global 0.5° grid to determine suitable habitat, resulting in a relative "probability of occurrence" for each species (S1B Fig). To roughly constrain species ranges to appropriate georegions, the AquaMaps model uses Food and Agriculture Organization of the United Nations (FAO) Major Fishing Area [16] boundaries. Of the resulting maps, 1,296 (5.7% of the full dataset) have been further refined through an expert review process that allows fine-tuning of input parameters to correct for known biases in sampling distributions of marine biological data (e.g. over-representation of shallower water depths, species misidentifications), and thus improves the range predictions [17,18].

In total, the two datasets provide range maps for 24,586 species, with a small subset of species mapped in both datasets. For the purposes of this analysis, we elected not to use IUCN data for bird species, which are available separately through Bird Life International [19].

#### Comparison of taxonomic and regional distribution

To examine the overall taxonomic distribution across the spatial datasets, we grouped species by taxonomic class and data source, and determined the proportion of each class represented in each dataset. To compare the spatial representation of the two datasets directly, we rasterized the IUCN species polygons to the same 0.5° grid as the AquaMaps species maps (S2 Fig). We determined species presence within a grid cell as any non-zero overlap of a species polygon with the cell (S1C Fig). For the AquaMaps dataset, we determined per-cell species count by including all species with non-zero probability of occurrence to best approximate the "extent of occurrence" generally indicated by IUCN maps (S1D Fig).

#### Comparison of paired maps

Although relatively small in number, overlapping species present a unique opportunity to evaluate the two datasets overall. For the species included in both datasets, we examine how well the maps align in both spatial distribution and overall area. If each dataset is communicating its own prediction of extent of occurrence, we expect that for a given species, the two predicted distributions will largely overlap, with similar total range sizes. Where these expectations seem to fail, we explore methodological issues that can introduce errors.

We must note that a close alignment between the two predicted distributions for a given species does not necessarily imply that the predictions are “correct” or even “good”; a close match could simply indicate that both datasets introduced similar errors of commission or omission. However, since the two datasets use distinct and independent methods to extrapolate range, in places where both datasets arrive at the same conclusion about species presence, it seems reasonable to assume a higher confidence in the species being present than in places where the two disagree.

We identified "paired map" species using IUCN Red List species identification codes, where available in both datasets, and otherwise relied on genus and species binomials as a matching key. To improve matching by name, we used rOpenSci’s “worrms” package [20] in R [21] to standardize spellings of species names and synonyms. For species with separate subpopulation maps in IUCN, we combined all subpopulations to create a single global population. For each of these paired map species, we determined species presence within each spatial cell for each dataset using the same criteria outlined above.

Overlaying paired distribution maps for each species, we defined and calculated *distribution alignment α*_*dist*_ and *area ratio α_area_*:

$$\alpha_{dist}=\frac{A_{small\cap large}}{A_{large}}*100\%$$

$$\alpha_{area}=\frac{A_{small}}{A_{large}}*100\%$$

For each paired map species, *A*_*small*_ and *A*_*large*_ indicate the smaller and larger range representation (regardless of which dataset) in km^2^. *A*_*small*∩*large*_ represents the amount of overlapping area between the two datasets. Distribution alignment uses overlapping predictions of presence as a measure of concurrence between the two datasets. Area ratio compares range size, used by IUCN as a criterion to help define extinction risk; it also provides an indicator for frequency of errors of commission (false indication of presence) or omission (false indication of absence).

#### Examining issues in paired map alignment

Given the wide variation in the alignment of predicted range for the paired map species, we examined two potential drivers of error, one for each dataset. For IUCN data, we explored the assumed consideration of depth as a criterion for range predictions of coral species, a large portion (14%) of the IUCN dataset and an intensely studied taxonomic group whose importance in supporting biodiversity is undisputed. Extracting data from the IUCN API [2] we identified depth limitations of each coral species mapped in the IUCN dataset to verify that none is is indicated to occur below the photosynthetic depth limit of 200 m. Using the same methodology as shown in S2 Fig, we created a 200 m bathymetry raster from a bathymetry spatial dataset (public domain; available from Natural Earth, www.naturalearthdata.com) and masked our IUCN coral rasters to identify mapped coral presence below 200 m. The resulting maps were again compared to the AquaMaps ranges to examine distribution alignment and area ratio.

For AquaMaps data, whose reliance upon FAO Major Fishing Areas [16] to constrain species ranges seems likely to result in abrupt discontinuities in species range where ecologically suitable habitat intersects with human-defined borders, we identified FAO boundaries where such discontinuities are likely to occur. Cropping species distributions to narrow latitudinal bands, we determined species whose eastern or western range limit coincided exactly with a defined FAO boundary. As an illustrative example, we cropped AquaMaps range maps for all Pacific and Indian Ocean species to a band between between 25° S and 20° N latitudes, then identified all species whose eastern range limit within this band coincided with 175° W longitude, the vertical boundary between two prominent FAO regions.

### Methods for MPA gap analysis case study

To assess the effectiveness of MPAs in protecting biodiversity, Klein et al. [13] compared the coverage of the global MPA network presented by the World Database on Protected Areas (WDPA) [22] to the species ranges described in the AquaMaps dataset, version 08/2013 [23]. For the primary analysis, the researchers defined species presence as 50% or greater probability of occurrence.

To reconstruct the primary analysis, we selected the subset of protected areas from the 2014 WDPA dataset classified as IUCN protected area management categories I-IV and spatially overlapping a marine area as per the original study. The WDPA polygons and marine polygons were rasterized to 0.01° and then aggregated to AquaMaps native 0.5° cells, to calculate proportion of marine protected area within each cell. After verifying our method using the 08/2013 AquaMaps dataset, we updated the analysis to use the 2015 AquaMaps dataset [1], at a presence threshold of 50% (to compare to Klein et al. directly) and 0% (to better compare with IUCN spatial data). To analyze MPA coverage against IUCN spatial data, we extracted IUCN polygon weights per 0.5° cell for each species and compared against the protected area raster.

All processing was completed using R statistical software [21], and all code and intermediate data are available on GitHub at https://github.com/OHI-Science/IUCN-AquaMaps. All maps were generated using public domain data from Natural Earth, www.naturalearthdata.com.

## Results and discussion

In comparing the IUCN and AquaMaps datasets, it is again important to emphasize that the two differ in both methodology and intent. For any given species, the IUCN range map and AquaMaps distribution (including all non-zero probability of occurrence) both effectively represent a prediction of extent of occurrence; therefore, we should expect the two maps to show significant overlap in predicted range, and to capture a similar total area. However, AquaMaps range maps are created independently from IUCN data and therefore exceptions are certain to arise. Here we are looking for systematic deviations from our expectations that might highlight implications of data use decisions.

### Taxonomic and geographic coverage

**Fig 1:**
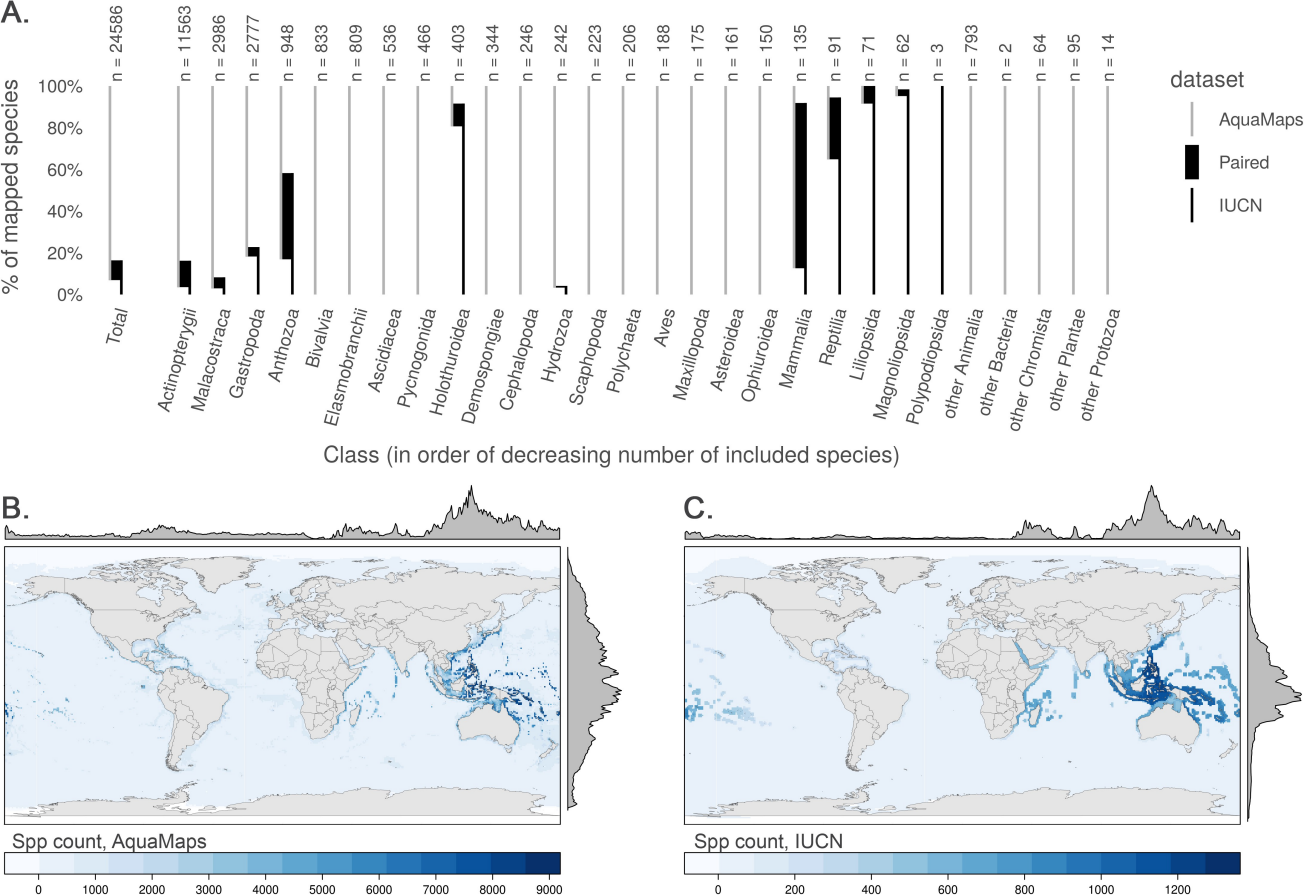
Taxonomic and geographic coverage of AquaMaps and IUCN range data. (A) Number and proportion of species by taxa included in each dataset (22,889 species in AquaMaps, 4,027 species in IUCN). Overlapping species are dominated by bony fishes (994 species, primarily tropical taxa) and corals (394 species). (B, C) Global marine species count per 0.5° cell according to (B) AquaMaps and (C) IUCN. The margin frequency plots show relative species count per cell at each latitude and longitude.

The two datasets have notably different taxonomic (Fig 1A) and regional (Fig 1B and 1C) coverage. AquaMaps encompasses a broader range of taxa than IUCN, as IUCN spatial data are only available for select taxonomic groups that have been comprehensively assessed. Of the 24,586 species mapped within these datasets, only 2,330 (9.5%) are mapped within both, with many taxa completely unrepresented in one dataset or the other. While species numbers in both datasets peak in tropical latitudes near the equator, species counts for IUCN maps drop quickly beyond 30°N and 30°S, while AquaMaps includes distribution of species well into temperate latitudes. Together, the limitations of spatial and taxonomic coverage are likely to drive a researcher's choice of dataset far more strongly than the quality, format, or intended purpose of the dataset.

### Distribution and range size alignment

**Fig 2:**
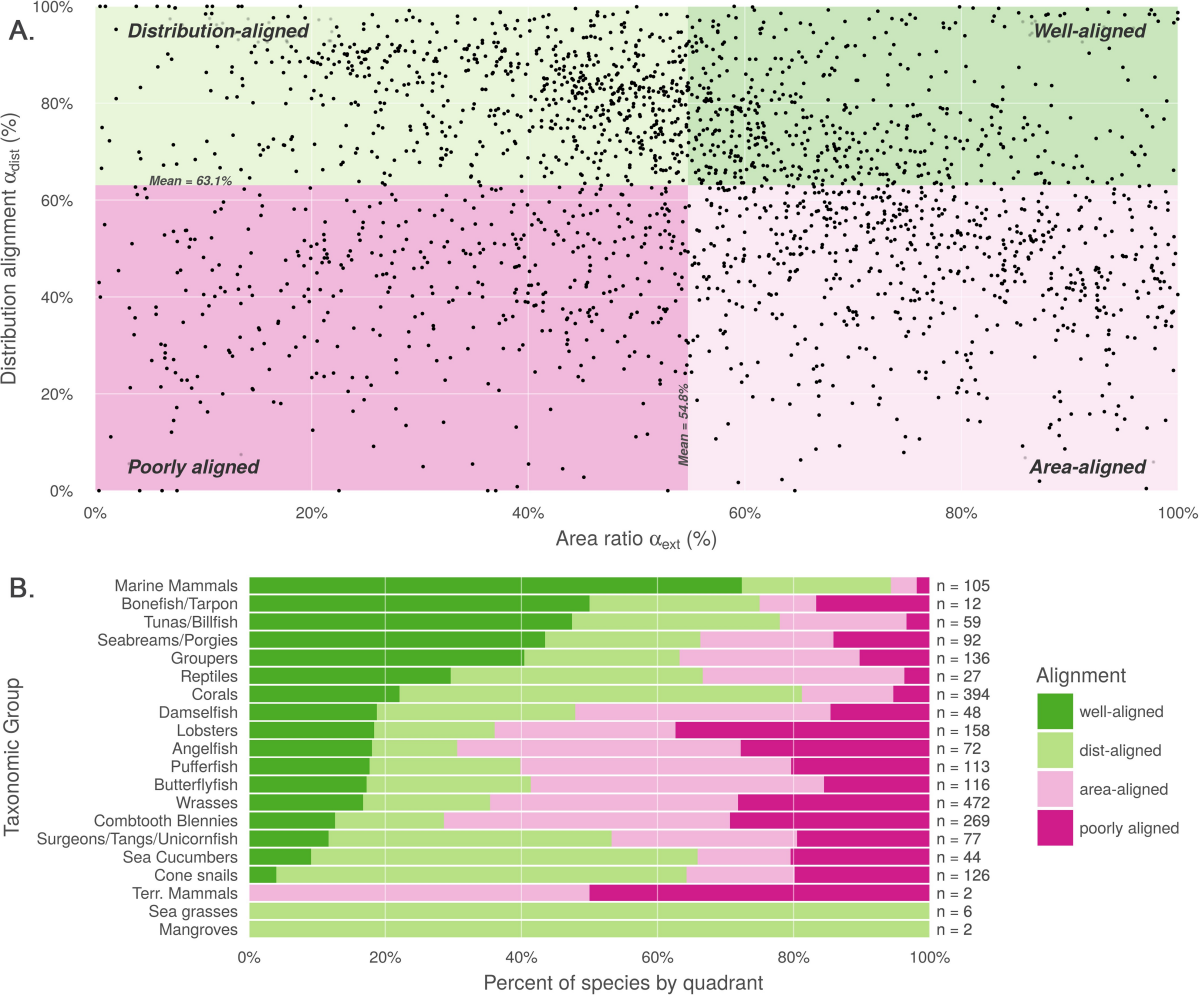
Comparison of alignment between AquaMaps and IUCN range data. (A) Distribution alignment (overlap of smaller range within larger) versus area ratio (the ratio of smaller range area to the larger range area) for 2,330 species included in both IUCN and AquaMaps datasets. The upper right quadrant comprises species whose maps largely agree in both spatial distribution and the extent of described ranges (n = 522; 22.4% of paired map species). The upper left quadrant comprises species whose maps agree well in distribution, but disagree in area (n = 715; 30.7%). The lower right quadrant includes species for which the paired maps generally agree in range area, but disagree on where those ranges occur (n = 649; 27.9%). The lower left quadrant indicates species for which the map pairs agree poorly in both area and distribution (n = 444; 19.1%). (B) Alignment quadrant breakdown of species by taxonomic group.

Comparing distribution alignment and area ratio for the 2,330 paired map species (Fig 2A), a weak negative linear pattern appears to emerge, suggesting that increasing similarity in range area correlates very slightly with decreasing distribution alignment (R^2^ = .016). The pattern itself is not particularly important, and emerges simply due to the nature of the analysis and the datasets. In particular, the AquaMaps model tends to extrapolate species ranges into suitable areas beyond known occurrences more frequently than IUCN maps, such that each additional unit of range predicted by AquaMaps will fall in different locations than an additional unit of range predicted using IUCN methodology. For species with range areas that were estimated to be very different in terms of size based on the different methodologies, predicted distribution for the smaller range can more easily fall within the generous bounds of the larger range. For species with increasingly similar range areas, differences in methodology become more difficult to "hide," and the distribution alignment generally becomes slightly poorer.

The mean distribution alignment for species included in both datasets was 63.1%; the mean area alignment was 54.8%. By dividing the paired map species into quadrants based on these means, we highlight categories of relationships to identify patterns in alignment differences. Representative maps from each category are provided in the supporting materials (S3 Fig).

The upper right quadrant includes the species (n = 522) whose described ranges are above average in alignment of both spatial distribution and area. These species tend to be well-studied and include wide-ranging pelagic organisms such as marine mammals, tunas, and billfishes (Fig 2B). This result is not surprising, as species with very large ranges are likely to be more aligned regardless of methodology simply because both predicted ranges span nearly the entire map.

The area-mismatched ranges contained in the upper left quadrant (n = 715) include many species whose spatial distribution is similar, but where one range is notably larger than the other. For 88.1% of the species in this quadrant, the IUCN range is larger than the AquaMaps range, which suggests a systematic introduction of commission errors by IUCN and/or omission errors by AquaMaps. Below we explore one underlying source of commission errors.

Species found in the lower right quadrant (n = 649) represent cases of "two wrongs make a right." For these species, IUCN and AquaMaps both predict ranges extending far beyond the overlapping region, but the methodological differences result in very different extrapolations. Consequently, area ratios are high, though the poor distribution alignment indicates that one or both datasets are introducing significant errors. In this quadrant, the IUCN range is the larger for only 56.5% of species, which does not seem to imply a systematic introduction of errors.

The lower left quadrant includes species (n = 444) where alignment is poor in both dimensions. In this quadrant, the IUCN range is larger for only 24.6% of species, suggesting a systematic introduction of commission errors by AquaMaps and/or omission errors by IUCN. Data-poor species are more common in this quadrant; indeed, the median number of species occurrence records (averaging occurrences from the Ocean Biogeographic Information System (OBIS) [24] and the Global Biodiversity Information Facility (GBIF) [25]) for this quadrant is 38 records, compared to a median of 229 records for species across the other three quadrants. The AquaMaps dataset offers its own quality metric based on the number of unique 0.5° cells containing valid occurrences; for this quadrant, the median "occurcells" is 10 compared to a median of 41 across the other three quadrants. Care should be taken when using distribution and range maps based upon fewer observations, as they clearly bear greater uncertainty; AquaMaps explicitly warns against using any of its maps generated with an "occurcells" count fewer than 10 [1].

It is unsurprising that species with expert-reviewed AquaMaps fare far better in this paired-map analysis; for expert-reviewed maps, the mean distribution alignment improved to 75.2% (compared to 63.1% for the full set of paired maps), while the mean area alignment improved 61.0% (compared to 54.8%). See S4 Fig for a version of Fig 2A and 2B focused on expert-reviewed species.

### Coral depth exploration

Because corals dominate the upper-left "distribution-aligned" quadrant of Fig 2A (n = 233; 32.6% of all species in this quadrant), we explored implications of explicitly restricting IUCN ranges to depths based on species' life histories. This adjustment was not necessary for AquaMaps data because models explicitly include ocean depth preference as a parameter. While depth is recommended by the IUCN as a criterion for providers of range maps ("The limits of distribution can be determined by using known occurrences of the species, along with the knowledge of habitat preferences, remaining suitable habitat, elevation limited, and other expert knowledge of the species and its range." [2]), it is not presented as a requirement, so we cannot take its inclusion for granted. Additionally, IUCN Red List mapping standards formerly required, and still allow, a 50 km buffer around the coastline for coastal species [26]; such a buffer directly conflicts with habitat limitations (such as depth) and distorts our understanding of species distribution.

**Fig 3:**
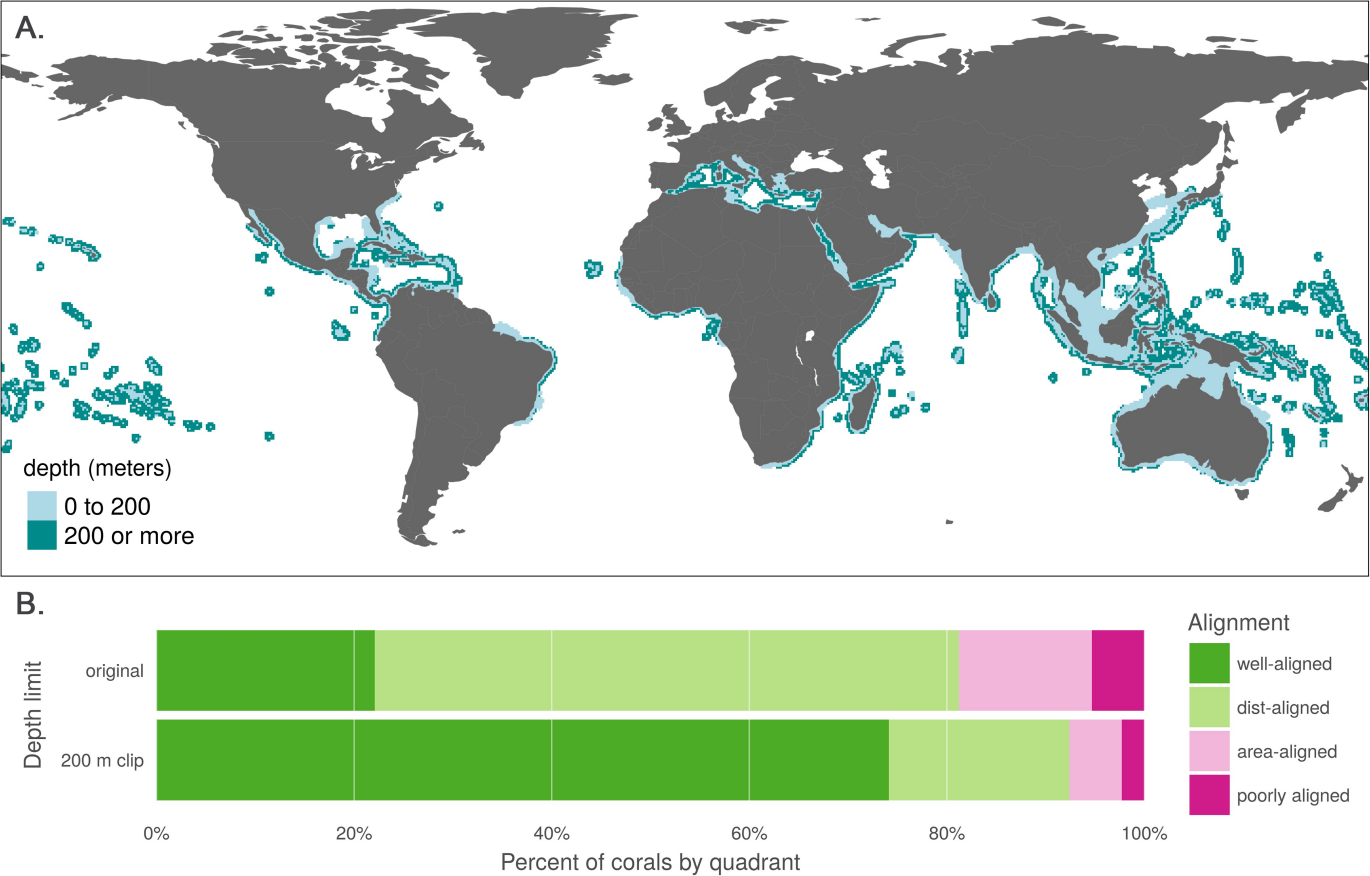
Effect of 200 m depth constraint on IUCN range maps for coral species. (A) Aggregate map combining ranges of the 562 coral species mapped in the IUCN dataset, showing raw ranges and ranges clipped to 200 m depth. (B) Alignment quadrant breakdown of paired map coral species using original data from IUCN and AquaMaps (as in Fig 2B) and the same species with IUCN ranges clipped to 200 m depth.

It must be noted that some coral species are known to occur at depths greater than 200 m, able to survive without photosynthetic zooanthellae. However, for the set of coral species included in IUCN range maps, IUCN habitat descriptions indicate that none occurs deeper than 200 m, and 94% are confined to waters shallower than 50 m; seven of the mapped species had no reported depth information. Clipping coral ranges to waters shallower than 200 m (Fig 3A) eliminated an average of 47.8% of the total predicted area while still allowing for a generous estimate of suitable habitat.

In constraining coral ranges to appropriate depths, we see a strong increase in the apparent alignment of species maps between IUCN and AquaMaps (Fig 3B). Membership in the "well-aligned" quadrant jumped from 22.1% to 74.1%, with a corresponding decrease in all other quadrants. By excluding the unsuitable areas from IUCN's predicted range, we eliminate preventable commission errors and more closely approximate the range described by AquaMaps. See S5 Fig to examine the shifts of individual species among the quadrants.

The true distribution of each of these corals remains imperfectly known. Certainly some commission errors result from IUCN Red List mapping standards including coastline buffers and “minimum convex polygons”, and others may be due to experts taking a precautionary (i.e., generous) approach to likely occurrence. Yet a simple and sensible shift in method drastically decreases the likelihood of introducing commission errors, with little chance of introducing omission errors, greatly improving our confidence in the remaining reported distributions for most purposes. This change applies just as readily to the IUCN coral maps that are not included in the paired map analysis, and likely to other coastal and reef-associated flora and fauna. While species depth preferences are an easy and consistent means of constraining range predictions, other conditions such as salinity and temperature could be cautiously used to refine the results of expert opinions, much as AquaMaps models use such conditions to predict suitable habitat.

### Georegional constraint exploration

**Fig 4:**
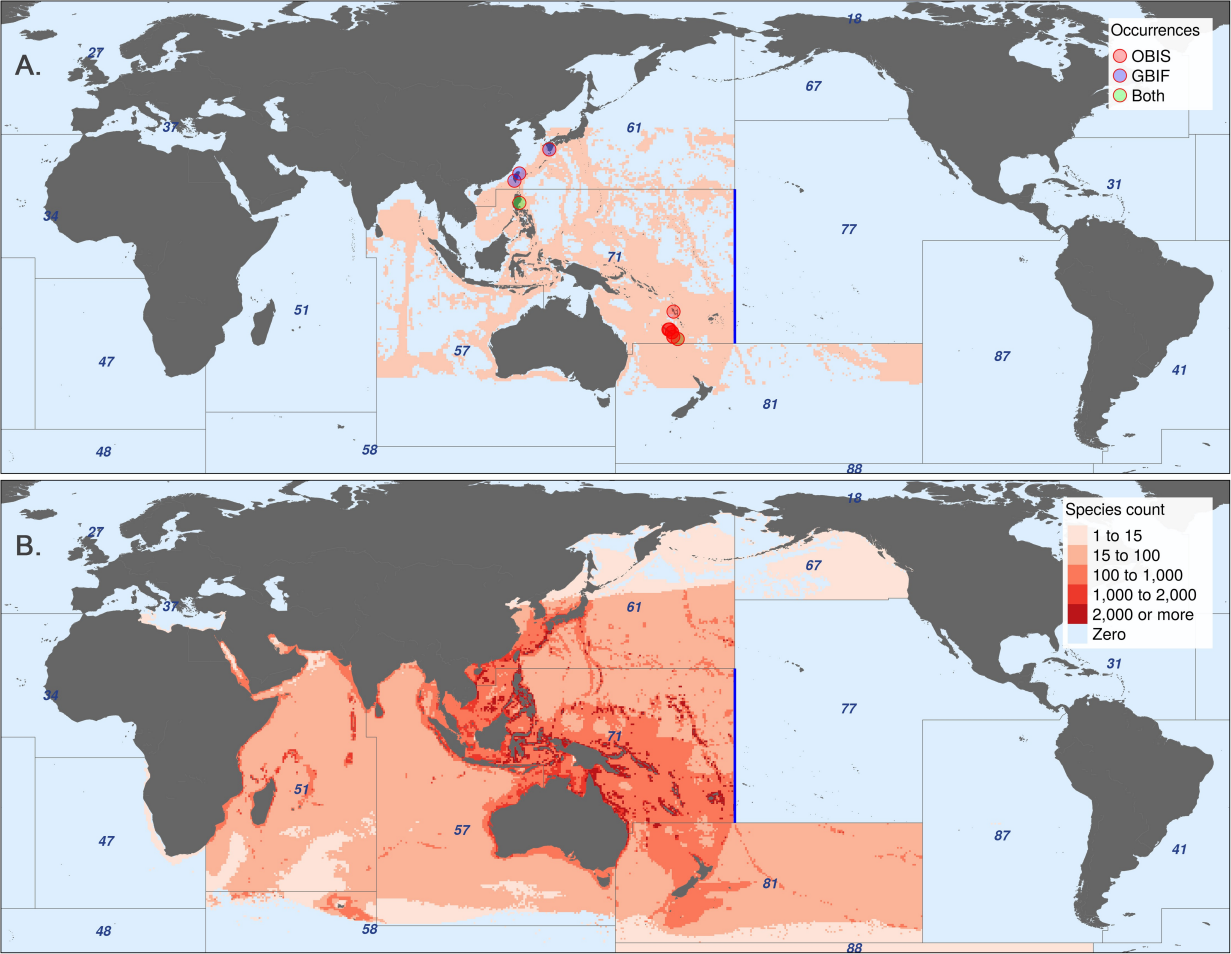
Effect of FAO Major Fishing Area constraints on AquaMaps distributions. (A) AquaMaps species distribution of *Hoplichthys regani*, the ghost flathead, with known occurrence records. (B) Aggregated AquaMaps predicted ranges for 3,208 species whose equatorial distribution encounters an eastern discontinuity exactly at 175° W, the boundary between FAO Major Fishing Areas 71 and 77 (shown in blue). Other FAO area boundaries create additional clear discontinuities.

From the entirety of the AquaMaps dataset, we identified 3,208 Indo-Pacific species whose equatorial distributions (between 25° S and 20° N) encounter an eastern range limit at 175° W. A clear discontinuity in species distributions of a single example species (Fig 4A) and all 3,208 species in aggregate (Fig 4B) matches perfectly with FAO region 77 [16]; other discontinuities are apparent at other FAO boundaries, despite these boundaries not being actively studied in this analysis.

FAO Major Fishing Area boundaries provide a readily available method to roughly constrain AquaMaps predictions to appropriate ocean basins, thus eliminating a large source of potential commission errors and enabling rapid modeling of thousands of species ranges. However, these boundaries are defined for statistical purposes based on economic and political considerations rather than ecological considerations, and can result in odd discontinuities in species range predictions where otherwise suitable habitat is excluded. While such a discontinuous boundary would likely be obvious when inspecting the distribution of an individual species, the distinction is likely to be obscured when aggregating many species ranges as is typical for biodiversity or conservation studies.

The ratio of the total predicted range for a species to the number of "occurcells" used to generate the map provides a measure of the degree to which AquaMaps extrapolates range area from limited data. For example, AquaMaps predicts a total range of 5.4 million km^2^ for both the round ray *Rajella fyllae* and the brittle star *Ophiothrix plana*; but the map for *R*. *fyllae* is generated using 116 "occurcells" (for a data extrapolation rate of 46,800 km^2^ per cell) while the map for *O*. *plana* is generated using only four (for an extrapolation rate of 1,360,000 km^2^ per cell).

By this measure, the 3,208 species range maps included in Fig 4 tend to extrapolate farther based on limited data: a mean predicted range of 827,200 km^2^ per "occurcell" compared to a mean predicted range of 485,300 km^2^ per "occurcell" for the overall AquaMaps dataset. This suggests that the FAO boundaries may not be sufficient to adequately constrain computer-generated ranges. To reduce the incidence of commission errors due to aggressive extrapolation, it may be desirable to fine-tune the computer model output with additional filters, such as ecoregional constraints, e.g. Marine Ecoregions of the World [27], or distance-based methods, e.g. inverse distance weighting to enforce proximity to known observations. Expert review, though time-consuming, is the most certain route to boosting confidence in these predicted distributions.

### Case Study: MPA gap analysis

Klein et al. [13] compare the global distribution of species to the global distribution of marine protected areas to assess how well current MPAs overlap with species ranges and identify which species fall through gaps in protection. The study relied on the 08/2013 version of the AquaMaps database [23], using a probability of occurrence threshold of 50% or greater, to determine species presence, and the World Database of Protected Areas [22] to define zones of marine protection. They found that the global MPA network leaves 90.5% of marine species with less than 5% of their overall range represented within MPAs, and 1.4% of species have no protection at all (i.e., "gap" species). But what if the researchers had chosen to use IUCN data for their analysis rather than AquaMaps?

**Fig 5:**
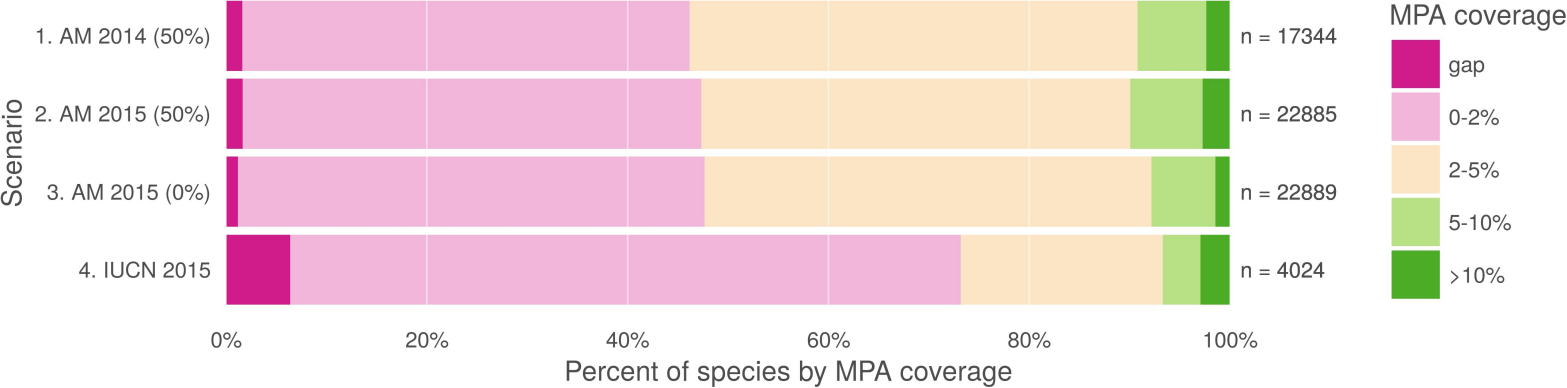
MPA gap analysis results based upon alternate choices of datasets. Percent of species range covered by MPAs based upon methods in Klein et al. (2015). Scenario 1 replicates the original results, measuring protected range of species in AquaMaps version 08/2013 dataset, with a 50% presence threshold, against the 2014 World Database of Protected Areas, filtered for IUCN categories I-IV that overlap marine areas. Scenario 2 updates the results using AquaMaps version 08/2015, showing very small changes despite the inclusion of an additional 5,545 species. Scenario 3, still using 2015 AquaMaps data, drops the presence threshold to zero, showing an expected decrease in gap species, but also a decrease in species with 5% or greater protected range. Scenario 4 examines species MPA coverage using only the IUCN dataset.

We recalculated the amount of under-protected and gap species using all available IUCN species ranges, as well as the 2015 AquaMaps data at a 50% threshold to replicate the original methods and a 0% threshold to more closely approximate the extent of occurrence represented by IUCN data (Fig 5). Comparing the IUCN results to the AquaMaps 2015 results (at 0% threshold) we found a five-fold increase in the proportion of gap species (6.4% of species vs. 1.2%) and dramatically larger proportion of species with less than 2% of their range protected (73.2% of species vs. 47.7%). However, this comparison also indicates a larger proportion of well-protected species with greater than 10% of range protected (2.9% of species vs. 1.5%).

In performing this analysis, our intent is not to call into question the assumptions, methodology, or results of the original MPA gap analysis. Rather, we intend to illustrate how differences between these two datasets could significantly influence conservation research outcomes (e.g. an apparent fivefold increase in gap species) and resulting conclusions about conservation efforts (e.g. the effectiveness of MPA policy).

## Conclusions

AquaMaps and IUCN range maps show strong agreement for many well-studied species, but for many others, substantial differences arise; unfortunately the methodological decisions that drive these differences (e.g. the use of FAO regions in AquaMaps and the 50 km coastal buffer in IUCN maps) are not readily apparent to novice or even experienced users of these datasets. While the burden ultimately falls upon data users to understand the limitations of the data, we hope our work will help the creators of these valuable datasets to communicate these limitations in a clear and concise way.

Researchers using these datasets may be able to take concrete steps to account for the tradeoffs we have highlighted. Trimming unsuitable habitat from the IUCN's extent of occurrence maps, for example by explicitly clipping them to appropriate depths for coastal and reef-associated species, would reduce commission errors introduced by adherence to IUCN mapping standards, particularly the legacy 50 km coastline buffer. Conversely, including all AquaMaps cells with a non-zero "probability of occurrence" (rather than using a probability threshold to determine presence, e.g. greater than 50% for the MPA gap analysis [13]) would allow for the most generous inclusion of species range, likely resulting in maps that more closely align with the intent of the IUCN's extents of occurrence.

Conclusions drawn from each of these datasets could paint dramatically different pictures of global marine biodiversity or the effectiveness of conservation management decisions. By highlighting the distinctions between these two important marine species range datasets, we hope to encourage stronger collaboration between taxa experts and species distribution modeling researchers in order to benefit both datasets. Combining the transparency of AquaMaps documentation of parameter settings and assumptions for computer-generated species distribution maps with the on-the-ground knowledge gained through IUCN’s expert-opinion based process could be a meaningful step towards a more transparent and reproducible approach for describing species ranges. By incorporating the best aspects of both distribution modeling and expert opinion we may ultimately be able to improve our ability to inform strategic and effective conservation policy that supports a resilient ocean ecosystem.

## Supporting information

S1 Fig Examples of AquaMaps and IUCN raw species range data for *Thunnus alalunga* (Albacore Tuna). (A) IUCN species distribution represented as extent of occurrence polygons. (B) AquaMaps species distribution represented as varying probabilities of occurrence assigned to 0.5° grid cells. (C) IUCN and (D) AquaMaps distributions recalculated to represent presence within 0.5° grid cells. See S1 File for reference information.
(TIF)

S2 Fig Rasterizing shapefiles provided by IUCN. A portion of the *T*. *alalunga* range map is used to exemplify the rasterization process. To enable direct comparison of IUCN species ranges to AquaMaps species ranges, the raw IUCN polygon (A) is overlaid with a 0.5° degree grid matching the AquaMaps grid (B). Each cell is assigned a value of "present" if the cell overlaps any portion of the polygon (C). The resulting raster (D). See S1 File for reference information.
(TIF)

S3 Fig Representative species maps to illustrate each quadrant from Fig 2A. Each map is positioned to match its quadrant in Fig 2A. FAO Major Fishing Area boundaries [16] are outlined in light grey. (A) Distribution-aligned: *Conus episcopatus*, the dignified cone snail. Distributions show excellent overlap in the western Pacific, though IUCN range extends well beyond the bounds of the AquaMaps range. (B) Well-aligned: *Kajikia albida*, the Atlantic white marlin. Distributions from each data set show nearly complete overlap, and very similar range size. (C) Poorly aligned: *Acanthurus nigroris*, the blue-lined surgeonfish. IUCN predicts species distribution only near the Hawaiian islands; AquaMaps predicts extensive distribution throughout the central and western Pacific Ocean. The datasets align in neither distribution nor range size. (D) Area-aligned: *Conus magnificus*, the magnificent cone snail. Distributions overlap in the southern Pacific, but align poorly elsewhere. The range sizes are similar. See S1 File for reference information.
(TIF)

S4 Fig Improvement in alignment due to expert review of AquaMaps. (A) Modification of Fig 2A to highlight species with expert-reviewed AquaMaps shows that the mean distribution alignment and mean area ratio both improve. (B) Including only expert-reviewed species in each quadrant shows increased membership in the well-aligned and distribution-aligned quadrants relative to Fig 2B.
(TIF)

S5 Fig Shift in alignment of paired-map coral species due to clipping IUCN ranges to areas shallower than 200 m. The grey lines represent the change in apparent alignment for a single species. Most coral species shift rightward from the upper left quadrant to the upper right, improving in area alignment with little if any loss in distribution alignment, since in general, only unsuitable habitat has been removed. Leftward shifts can be seen in species whose larger original range is represented in AquaMaps; by trimming IUCN ranges, the area ratio becomes smaller.
(TIF)

S1 File References for Supporting Figures. (DOCX)
