# Supplementary figures and images for: Aligning marine species range data to better serve science and conservation
Source: PLoS One. 2017 May 3;12(5):e0175739. doi: 10.1371/journal.pone.0175739 (PMC5414950; doi:10.1371/journal.pone.0175739)

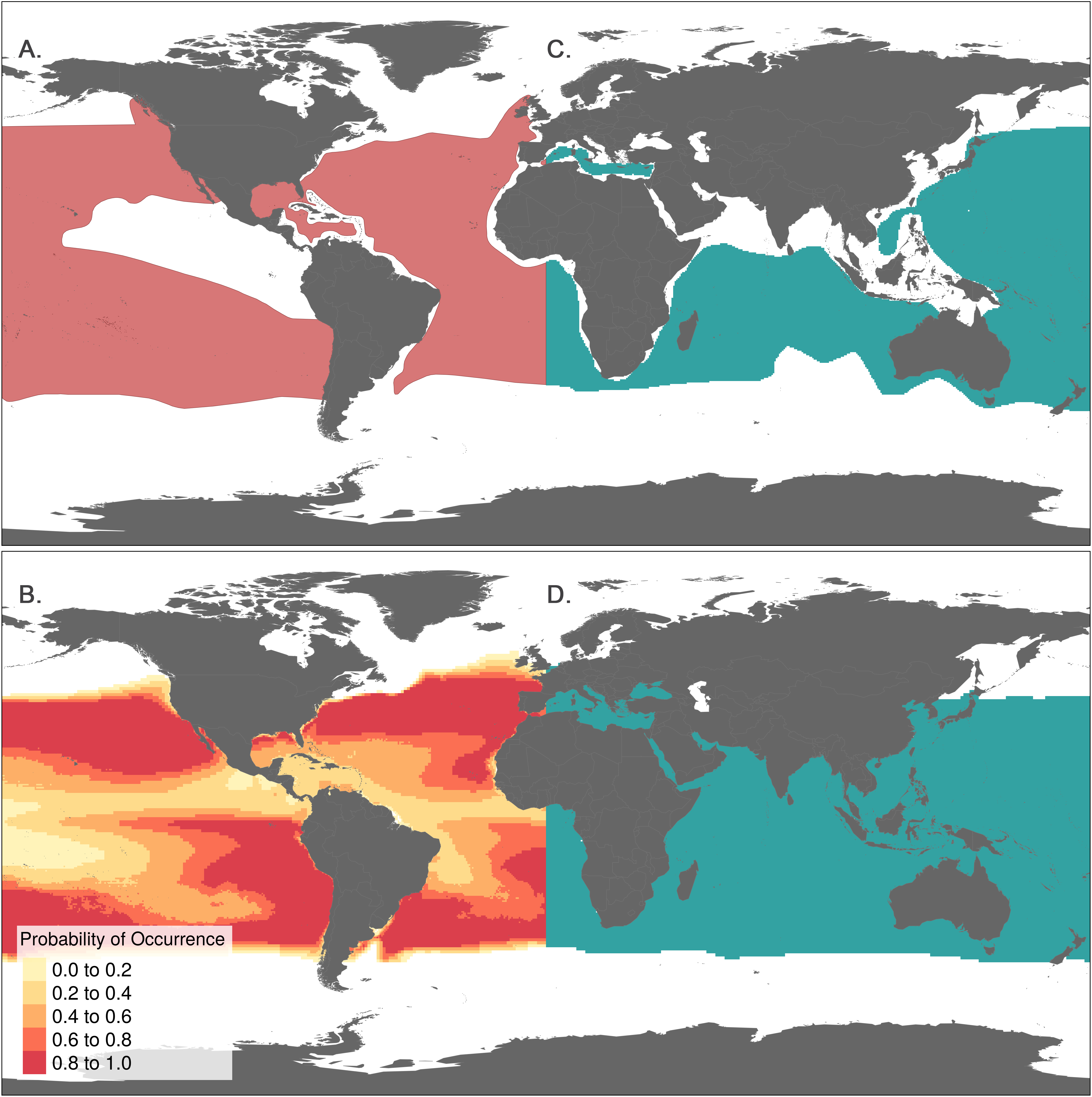

Supplement: S1 Fig — (A) IUCN species distribution represented as extent of occurrence polygons. (B) AquaMaps species distribution represented as varying probabilities of occurrence assigned to 0.5° grid cells. (C) IUCN and (D) AquaMaps distributions recalculated to represent presence within 0.5° grid cells. See S1 File for reference information. (TIF) [file pone.0175739.s001.tif]

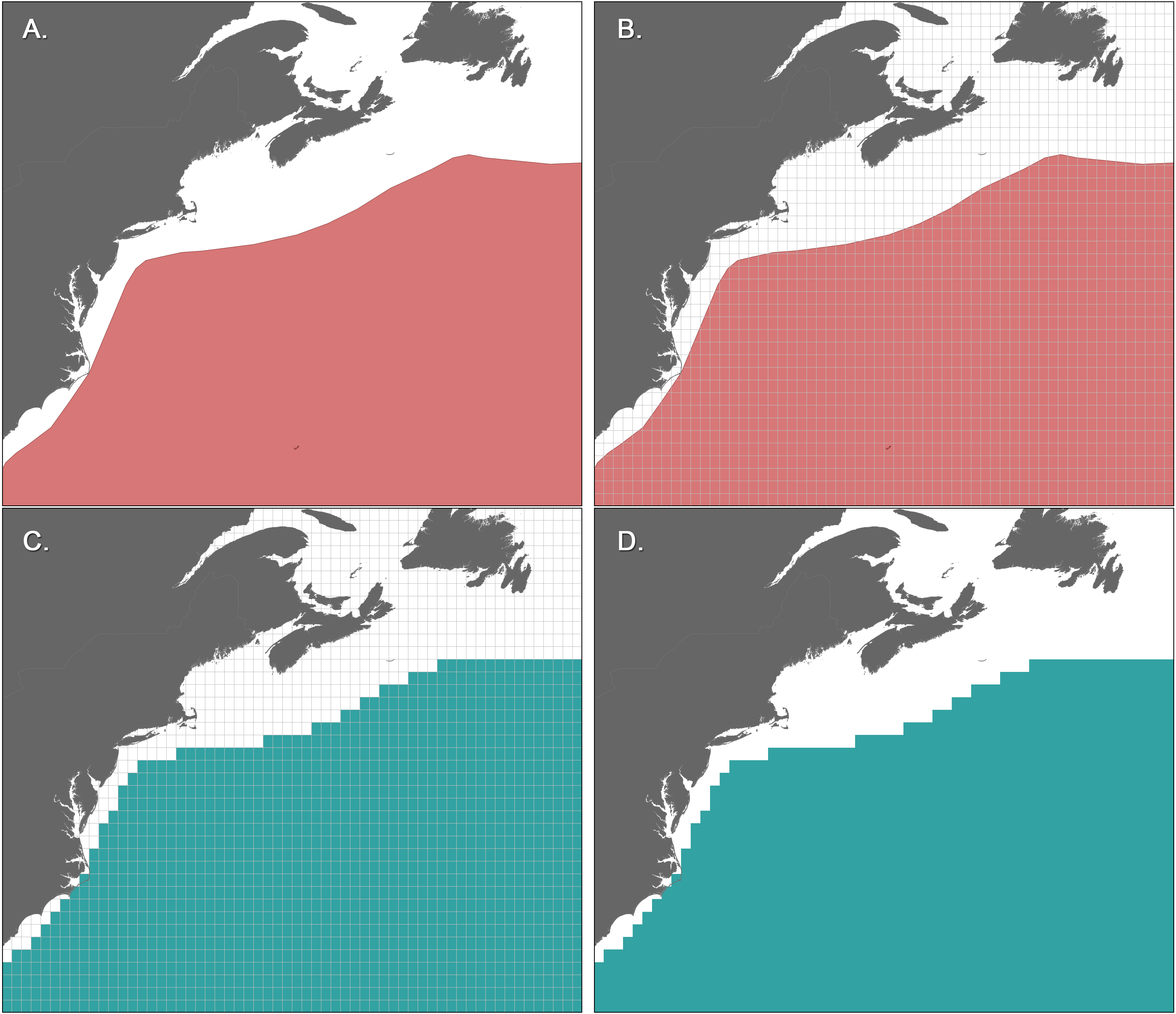

Supplement: S2 Fig — A portion of the T. alalunga range map is used to exemplify the rasterization process. To enable direct comparison of IUCN species ranges to AquaMaps species ranges, the raw IUCN polygon (A) is overlaid with a 0.5° degree grid matching the AquaMaps grid (B). Each cell is assigned a value of "present" if the cell overlaps any portion of the polygon (C). The resulting raster (D). See S1 File for reference information. (TIF) [file pone.0175739.s002.tif]

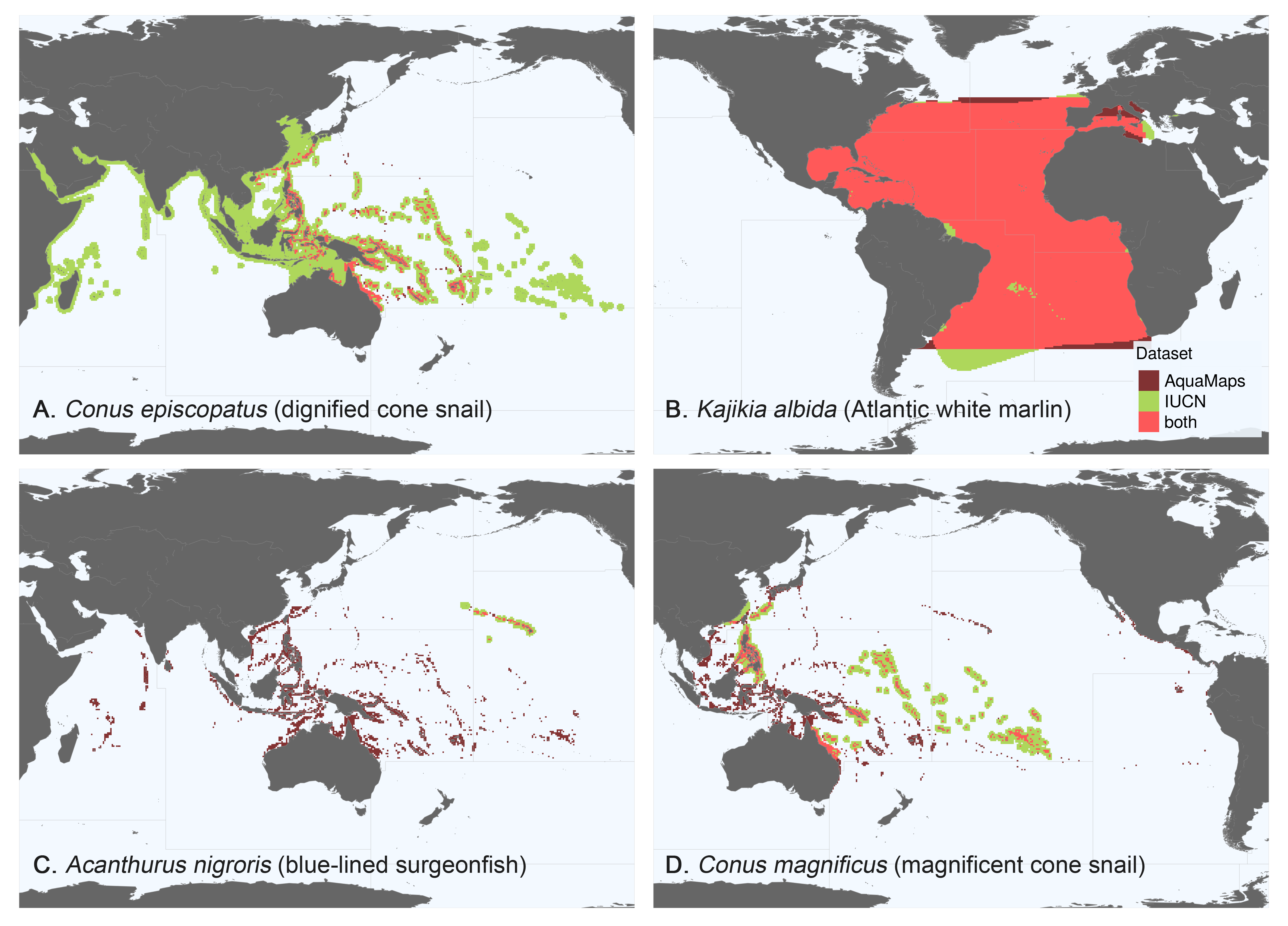

Supplement: S3 Fig — Each map is positioned to match its quadrant in Fig 2A. FAO Major Fishing Area boundaries [16] are outlined in light grey. (A) Distribution-aligned: Conus episcopatus, the dignified cone snail. Distributions show excellent overlap in the western Pacific, though IUCN range extends well beyond the bounds of the AquaMaps range. (B) Well-aligned: Kajikia albida, the Atlantic white marlin. Distributions from each data set show nearly complete overlap, and very similar range size. (C) Poorly aligned: Acanthurus nigroris, the blue-lined surgeonfish. IUCN predicts species distribution only near the Hawaiian islands; AquaMaps predicts extensive distribution throughout the central and western Pacific Ocean. The datasets align in neither distribution nor range size. (D) Area-aligned: Conus magnificus, the magnificent cone snail. Distributions overlap in the southern Pacific, but align poorly elsewhere. The range sizes are similar. See S1 File for reference information. (TIF) [file pone.0175739.s003.tif]

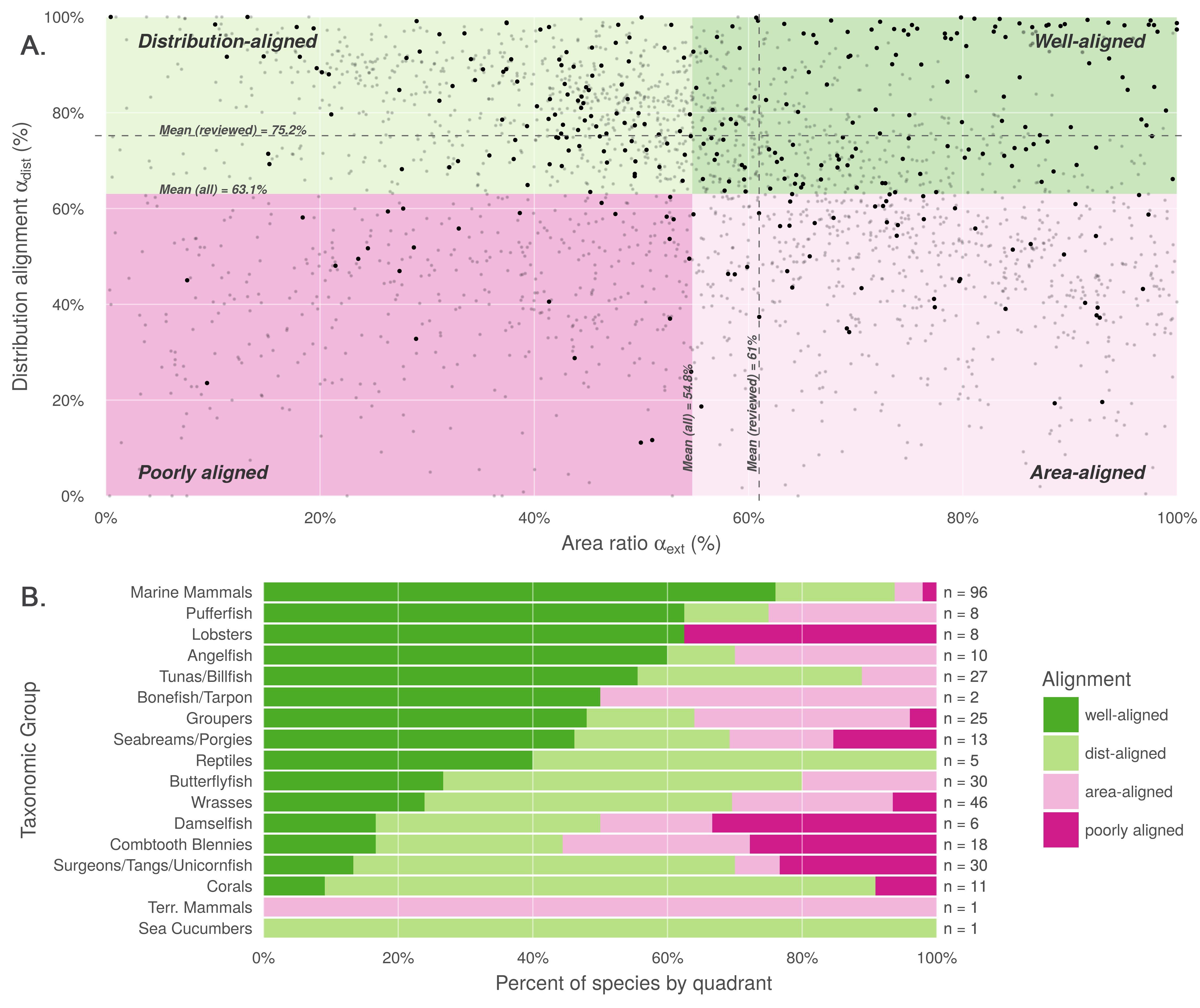

Supplement: S4 Fig — (A) Modification of Fig 2A to highlight species with expert-reviewed AquaMaps shows that the mean distribution alignment and mean area ratio both improve. (B) Including only expert-reviewed species in each quadrant shows increased membership in the well-aligned and distribution-aligned quadrants relative to Fig 2B. (TIF) [file pone.0175739.s004.tif]

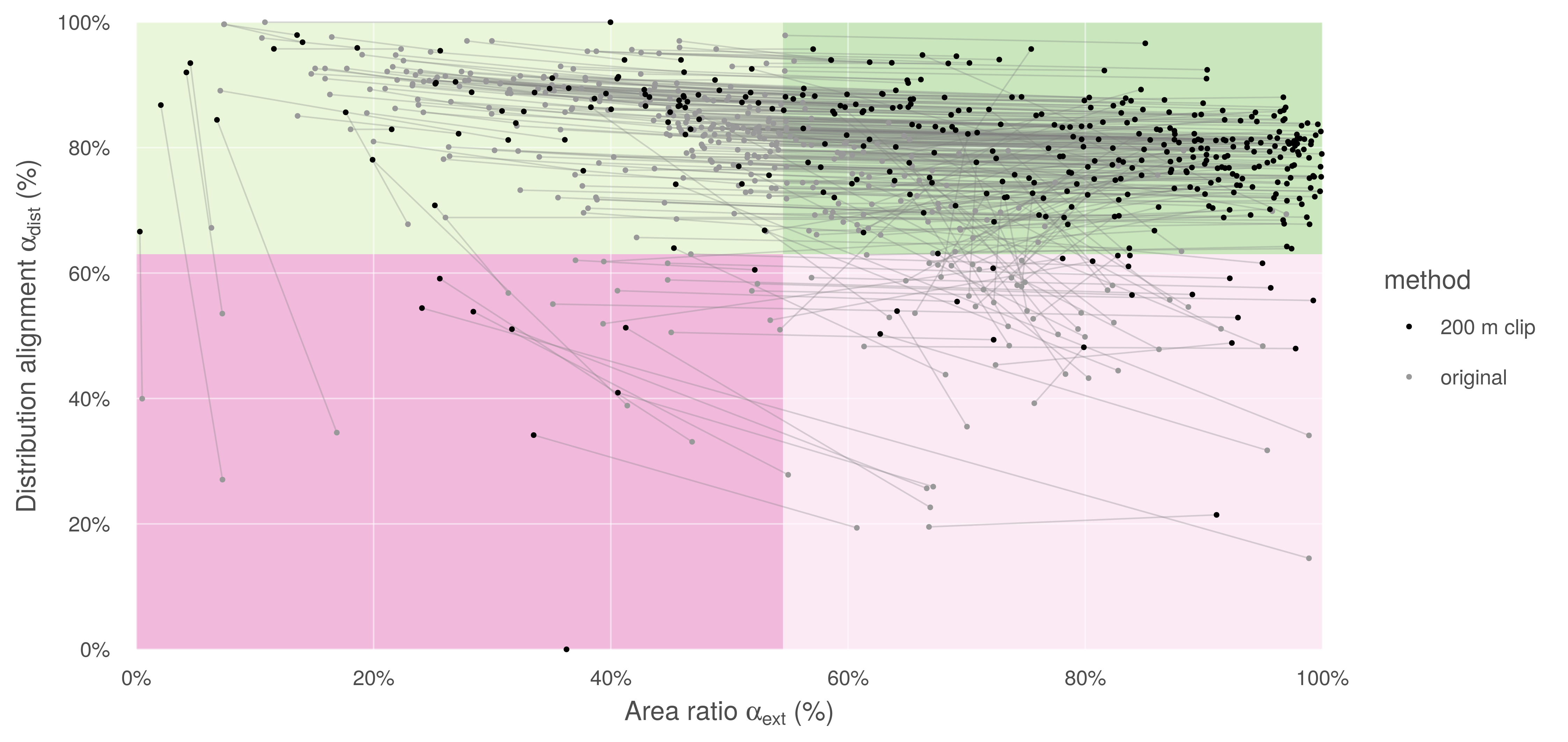

Supplement: S5 Fig — The grey lines represent the change in apparent alignment for a single species. Most coral species shift rightward from the upper left quadrant to the upper right, improving in area alignment with little if any loss in distribution alignment, since in general, only unsuitable habitat has been removed. Leftward shifts can be seen in species whose larger original range is represented in AquaMaps; by trimming IUCN ranges, the area ratio becomes smaller. (TIF) [file pone.0175739.s005.tif]
