# Supplementary material for: Aligning marine species range data to better serve science and conservation
Source: PLoS One. 2017 May 3;12(5):e0175739. doi: 10.1371/journal.pone.0175739 (PMC5414950; doi:10.1371/journal.pone.0175739)
Supplement: S1 File — (DOCX) [file pone.0175739.s006.docx]

Aligning marine species range data to better serve science and conservation

Casey O'Hara, Jamie Afflerbach, Courtney Scarborough, Kristin Kaschner, Benjamin S. Halpern

## References for supporting figures

**S1 Fig and S2 Fig references**

28. www.aquamaps.org, version of Aug. 2013. Reviewed distribution maps for Thunnus alalunga (Albacore), with modeled year 2100 native range map based on IPCC A2 emissions scenario. Web. Accessed 11 Sep. 2016.

29. International Union for Conservation of Nature (IUCN) 2011. Thunnus alalunga. The IUCN Red List of Threatened Species. Version 2016-2.

**S3 Fig references**

30. www.aquamaps.org, version of Aug. 2013. Computer generated distribution maps for Conus episcopatus, with modeled year 2100 native range map based on IPCC A2 emissions scenario. Web. Accessed 11 Sep. 2016.

31. Conch Books, Hackenheim, Germany 2013. Conus episcopatus. The IUCN Red List of Threatened Species. Version 2016-2.

32. www.aquamaps.org, version of Aug. 2013. Computer generated distribution maps for Kajikia albida (Atlantic white marlin), with modeled year 2100 native range map based on IPCC A2 emissions scenario. Web. Accessed 11 Sep. 2016.

33. International Union for Conservation of Nature (IUCN) 2011. Kajikia albida. The IUCN Red List of Threatened Species. Version 2016-2.

34. www.aquamaps.org, version of Aug. 2013. Reviewed distribution maps for Acanthurus nigroris (Bluelined surgeonfish), with modeled year 2100 native range map based on IPCC A2 emissions scenario. Web. Accessed 11 Sep. 2016.

35. International Union for Conservation of Nature (IUCN) 2012. Acanthurus nigroris. The IUCN Red List of Threatened Species. Version 2016-2.

36. www.aquamaps.org, version of Aug. 2013. Computer generated distribution maps for Conus magnificus, with modeled year 2100 native range map based on IPCC A2 emissions scenario. Web. Accessed 11 Sep. 2016.

37. Conch Books, Hackenheim, Germany 2013. Conus magnificus. The IUCN Red List of Threatened Species. Version 2016-2.
